# NFAT5 promotes oral squamous cell carcinoma progression in a hyperosmotic environment

**DOI:** 10.1038/s41374-020-00486-1

**Published:** 2020-09-08

**Authors:** Shohei Yoshimoto, Hiromitsu Morita, Miho Matsuda, Yoshinori Katakura, Masato Hirata, Shuichi Hashimoto

**Affiliations:** 1 Section of Pathology, Department of Morphological Biology, Division of Biomedical Sciences, Fukuoka Dental College, Fukuoka, Japan; 2 Oral Medicine Research Center, Fukuoka Dental College, Fukuoka, Japan; 3 The Center for Visiting Dental Service, Department of General Dentistry, Fukuoka Dental College, Fukuoka Dental College, Fukuoka, Japan; 4 Laboratory of Molecular and Cellular Biochemistry, Faculty of Dental Science, Kyushu University, Fukuoka, Japan; 5 Department of Bioscience and Biotechnology, Faculty of Agriculture, Kyushu University, Fukuoka, Japan

**Keywords:** Oral cancer, Cancer microenvironment

## Abstract

Epidermal growth factor receptor (EGFR) is highly expressed in several types of cancer cells including oral squamous cell carcinoma (OSCC). EGF/EGFR signaling is recognized as an important molecular target in cancer therapy. However, cancer cells often become tolerant to EGF/EGFR signaling-targeted therapies. In the tumor microenvironment, the tumor incites inflammation and the inflammation-derived cytokines make a considerable impact on cancer development. In addition, hyperosmolarity is also induced, but the role of osmotic stress in cancer development has not been fully understood. This study demonstrates molecular insights into hyperosmolarity effect on OSCC development and shows that NFAT5 transcription factor plays an important functional role in enhancing the oral cancer cell proliferation by inducing the EGFR translocation from the endoplasmic reticulum to the plasma membrane through increase the expression of DPAGT1, an essential enzyme for catalyzing the first committed step of N-linked protein glycosylation. These results suggest that hyperosmolarity-induced intra-nuclear translocation of NFAT5 essential for DPAGT1 activation and EGFR subcellular translocation responsible for OSCC tumor progression.

## Abstract (web summary)

This study demonstrates that NFAT5 promotes oral squamous cell carcinoma progression in the hyperosmotic environment through increased expression of DPAGT1, an essential enzyme for protein glycosylation, and altered EGFR subcellular localization from the cytoplasm to the plasma membrane in tumor cells.

## Introduction

Epidermal growth factor receptor (EGFR) is highly expressed in several carcinomas including oral squamous cell carcinomas (OSCC). EGF binds to the extracellular domain of EGFR and induces its dimerization, then intrinsic kinase activity is activated. Subsequently the intracytoplasmic domain of EGFR is autophosphorylated at multiple tyrosine residues. These phosphorylated tyrosine residues are bound to several adapter proteins, the EGF/EGFR signaling pathway is activated, which plays a critical role in cancer cells proliferation [1]. Thus, the EGF/EGFR signaling pathway is recognized as an important molecular target in cancer therapy. Some informative studies report the efficacy of EGFR-targeted therapies on cancer progression using EGFR tyrosine kinase inhibitors (TKIs) or EGFR-neutralizing antibodies [2, 3]. However, drug resistance sometimes occurs in these molecularly targeted therapies[4]. In addition, mutations of some genes such as *TP53*, *CDKN2A*, *PTEN*, *PIK3CA*, and *HRAS* were reported in OSCC [5, 6]. Therefore, it is essential to develop a new therapeutic strategy in addition to clarifying the mechanisms of anti-tumor drug resistance in molecularly targeted cancer therapies.

The cancer microenvironment is constantly inflamed and consist of cancer cells, connective tissues, vascular tissues and inflammatory cells [7]. It is a reasonable framework that inflammatory responses against cancer cells play an important role in cancer development [7, 8]. Several studies have shown that tumor associated macrophages play a pivotal role in cancer-related inflammation and progression of disease [9, 10]. Although in the cancer microenvironment, osmolarity is elevated under cancer cell-related inflammatory conditions, the relationship between hyperosmolarity and cancer cells proliferation remains unclear.

Under hyperosmotic conditions, nuclear factor of activated T cells 5 (NFAT5), a transcription factor, is involved in the regulation of cell homeostasis. NFAT5 is a transcription factor of the Rel family which also comprises the NF-κB and NFAT1-4 proteins [11]. NFAT1-4’s functional abilities are upregulated in a calcium/calcineurin-dependent manner by binding to calcineurin through their N-terminal calcineurin-binding domain site. In contrast, NFAT5 lacks the calcineurin-binding domain and is upregulated in a calcium/calcineurin-independent manner. NFAT5 is also known as tonicity-response element-binding protein and is expressed in variety of tissues. Its role is to control a genetic program to restore cellular homeostasis under hyperosmotic conditions [11, 12]. In addition, some previous reports have shown roles for NFAT5 in cancer tissues [13, 14], but the details of their functions, especially in cancer progression in the microenvironment, still remain unclear.

The aim of this study was to determine NFAT5 expression in the hyperosmotic OSCC tumor microenvironment and show how NFAT5 affected OSCC cell behavior, such as tumor progression.

## Materials and methods

### Cell cultures under normal and hyperosmotic conditions

The human tongue squamous cell carcinoma cell line HSC-3 was kindly donated by Dr. H. Takeuchi (Kyushu Dental University, Kitakyushu, Japan). HSC-4 was purchased from JCRB cell bank (Osaka, Japan). Cells were grown in Dulbecco’s Modified Eagle’s Medium (DMEM, Thermo Fisher Scientific, Waltham, MA), supplemented with 10% fetal bovine serum (FBS, PAA Laboratories, Pasching, Austria) at 37 °C in 5% CO_2_. In total, 25 mM glucose or 5.5 mM glucose contained DMEM was used as a high glucose (HG) or low glucose (LG) culture medium, respectively. To increase the osmolarity of medium, mannitol (Wako, Osaka, Japan) was added. The osmolarity of each solution was measured by osmometer (OM-801; Vogel GmbH & Co. KG, Fernwald, Germany). Cells were reseeded for the next passage after the trypsin (Thermo Fisher Scientific) dispersion when culture cells reached ~80% confluency.

### Western blot analyses

Cells were homogenized in an ice-cold lysis buffer and centrifuged for 30 min at 4 °C. The supernatants (20 μg) were separated by 8% sodium dodecyl sulfate-polyacrylamide gel electrophoresis (SDS-PAGE) and transferred to polyvinyldifluoride membranes (Millipore, Darmstadt, Germany). Immunoblot analyses were performed using the following primary antibodies; rabbit anti-EGFR and rabbit anti-phospho-EGFR (1:1000, Cell Signaling Technology, Danvers, MA), rabbit anti-NFAT5 (1:1000, Abcam, Cambridge, MA) and rabbit anti-DPAGT1 (1:1000, Sigma-Aldrich, St.Louis, MO) antibodies. A rabbit anti-human β-actin antibody (1:2000, Cell Signaling Technology) was used as an internal control. Blots were developed with horseradish peroxidase-linked secondary antibodies (1:3000, GE Healthcare, Cleveland, OH) and visualized by the enhanced chemiluminescence system using ImmunoStar Zeta (Wako), and the image density of bands were detected by LAS-4000 (GE Healthcare, Little Chalfont, UK). For the biotinylation assay, culture cells reached 80% confluency in a 10 cm diameter dish were lysed in the lysis buffer, and followed by an incubation in the cold biotin reagent (1 mg/ml sulpho-NHS-SS-biotin; Thermo Fisher Scientific) for 30 min at 4 °C. After the biotin-treated lysate was centrifuged at 18,000 × *g* for 10 min at 4 °C, the supernatant was incubated with 300 μL of cold avidin beads (Thermo Fisher Scientific) at 4 °C for 2 h and centrifuged at 3500 × *g* for 30 min at 4 °C. The supernatants were aspirated, and the beads were washed three times with 1 ml of the cold lysis buffer and once with a cold 500 mM NaCl and Tris-HCl (pH 7.5) buffer, and then once with a cold 10 mM Tris-HCl (pH 7.5) buffer. The beads were boiled with 50 μl of Laemmli sample buffer, and a 20-μl aliquot was analyzed by SDS-PAGE. For the deglycosylation, cell lysates were boiled in a 100 mM sodium phosphate buffer for 10 min in the presence of 2.5% 2-mercaptoethanol and 2% SDS. *N*-linked carbohydrates were removed by a treatment with 2 unites of N-glycosidase F (Roche, Basel, Switzerland) per 20 μg proteins. The mixture was incubated for 18 h at 37 °C. After the incubation, Western blotting analysis was carried out.

### Fluorescent immunocytochemistry

Cells were washed in a 1× phosphate buffered saline (PBS) (−) and fixed with a 4% paraformaldehyde (PFA) for 10 min at room temperature. After washing in a PBS (−), cells were permeabilized with digitonin (100 μg/ml, Wako) and blocked with 2.5% bovine serum albumin (BSA, Sigma-Aldrich). After lightly washing in a PBS (−), cells were incubated with the following each primary antibody; rabbit anti-EGFR antibody (1:100, Cell Signaling Technology), rabbit anti-NFAT5 antibody (1:100, Abcam), rabbit anti-DPAGT1 antibody (1:100, Sigma-Aldrich), mouse anti-KDEL antibody (1:100, MBL, Nagoya, Japan), for overnight at 4 °C. After three times washing in a PBS (−), cells were then incubated with the following each secondary antibody; Alexa Fluor 488 or 594-conjugated goat anti-rabbit or -mouse IgG (1:1000 dilution), for 1 h at room temperature. Nuclear counter staining was done with DAPI (1:3000 dilution) for 1 h at room temperature. After washing, the cells were mounted with PermaFluor mountant for visualization. Samples were visualized with a confocal microscope (LSM700, Carl Zeiss, Oberkochen, Germany). The images were processed in ZEN 2010B Sp1 Ver. 6.0.0.485 software (Carl Zeiss).

### Cell proliferation assays

The evaluation of the cell proliferation activity was done using the Cell Counting Kit-8 (WST-8, Dojindo, Kumamoto, Japan) and BrdU Proliferation Colorimetric ELISA Kit (Exalpha Biologicals, Shirley, MA) according to the manufacturer’s protocols. Briefly, cells were plated in 96-well plates at a ratio of 1 × 10^4^ cells/well and cultured in the LG or hyperosmotic conditioned medium with or without inhibitors, CP380736, a specific EGFR inhibitor, and genistein, a nonspecific TKI (Sigma-Aldrich), for 72 h at 37°C in 5% CO_2_. The culture cells in each treatment were incubated with 10 μl of tetrazolium substrate for 1 hr or 20 μl of BrdU for 6 h, then the absorbance was measured at a wavelength of 450 nm.

### *NFAT5* gene knockdown assays by short hairpin RNA (shRNA)

The oligonucleotides that contain the siRNA-expressing sequences targeting *NFAT5* (sh-*NFAT5*#1 top 5′-GATCCCCCAACATGCCTGGAATTCAATTCGAAGAGTTGAATTCCAGGCATGTTGTTTTTA-3′, bottom 5′-AGCTTAAAAACAACATGCCTGGAATTCAACTCTTCGAATTGAATTCCAGGCATGTTGGGG-3′; sh-*NFAT5*#2 top 5′-GATCCCCCCAGTTCCTACAATGATAATTCGAAGAGTTATCATTGTAGGAACTGGTTTTTA-3′, bottom 5′-AGCTTAAAAACCAGTTCCTACAATGATAACTCTTCGAATTATCATTGTAGGAACTGGGGG-3′) were annealed, and cloned into the pSUPER.retro.puro vector (OligoEngine, Seattle, WA). Viral supernatants were produced after transfection of 293 T cells with pGag-pol, pVSV-G and individual expression vector using the HilyMax (Dojindo). The cells were cultured at 37 °C in DMEM supplemented with 10% FBS for 24 h. The target cells were infected with each collected viral supernatant in the presence of 10 μg/ml polybrene (Merck-Millipore, Billerica, MA). After infection, the cells were cultured in the selection medium with 2 μg/ml puromycin (Enzo Life Science, Farmingdale, NY) for 48 h at 37 °C to get the cells showing *NFAT5* gene integration in their own genomic DNA.

### Real-Time-quantitative PCR (RT-qPCR) analyses

Total RNA was purified using RNeasy Mini Kit (Qiagen, Hilden, Germany), and cDNA was prepared using the ReverTra Ace (Toyobo, Osaka, Japan), according to the manufacturer’s protocols. RT-qPCR was performed using KOD SYBER qPCR Mix (Toyobo) and Thermal Cycler Dice Real Time System TP-960 (Takara, Shiga, Japan). When samples were analyzed, *NFAT5* or *DPAGT1* mRNA expression levels were normalized by the corresponding *GAPDH* mRNA expression level. The PCR primer sequences used were as follows: *NFAT5* forward 5′-AGGATGAGGGGTGTGGATTG-3′, reverse 5′-GCCTCTGCTTTGGATTTCGT-3′; *DPAGT1* forward 5’-GGGCGTTTCTTGCCCTCTAC-3′, reverse 5′-CCTGGCCCAAGTTCTATCCC-3′; *GAPDH* forward 5′-ATCACCATCTTCCAGGAGCGAG-3′, reverse 5′-TGGCATGGACTGTGGTCATG-3′.

### In situ Proximity Ligation Assay

In situ Proximity Ligation Assay (in situ PLA) was performed to investigate co-localization of proteins using Duolink™ (Sigma-Aldrich), according to the manufacturer’s protocol. In brief, cells were fixed with 4% PFA and incubated with a pair of primary antibodies directed to EGFR (Cell Signaling Technology) and to KDEL (MBL) in 2.5% BSA. This was followed by washing with a wash buffer and consecutive 1 h incubation with corresponding PLA probes, to which oligonucleotides secondary antibodies were conjugated (mouse minus and rabbit plus). The cells were washed and incubated with the ligation solution for 30 min. After washing with a wash buffer, the cells were treated with the polymerase for amplification for 100 min, and then mounted with the Mounting Media for visualization. Samples were viewed with a microscope (BZ-9000, KEYENCE, Osaka, Japan). Quantitative fluorescence cell image analysis was performed using the BZ-II Analyzer. exe ver. 2.1 (KEYENCE).

### Chromatin immunoprecipitation

Chromatin immunoprecipitation (ChIP) was performed on lysates from HSC-3 cells using the SimpleChIP Kits (Cell Signaling Technology) and ChIP grade anti-NFAT5 antibody (Abcam). Binding of NFAT5 to DPAGT1 promoter region was detected by PCR. The PCR primer sequences used were as follows: forward primer, 5′-GGAGGCATCCCAGATTAAGG-3′, and, reverse primer, 5′-GAGCGGAAATGCCAATTGCC-3′.

### Tumorigenicity analyses

HSC-3 cells (1 × 10^6^ cells) in 0.2 ml of PBS were injected subcutaneously into the right flank of the 8-week-old female BALB/cAJcl-nu mice (CLEA Japan, Inc, Tokyo, Japan) and maintained at the Center for Animal Resources of Fukuoka Dental College under specific pathogen-free (SPF) conditions. The mice were handled in accordance with the animal care policy of Fukuoka Dental College. After 1 week from the injection, a microosmotic pump (ALZET, model 1004; DURECT, Cupertino, CA) was implanted in the right flank to sustain the release of PBS or mannitol (289.5 μg/mouse/day) into the microenvironment of the transplanted HSC-3 cells. Each tumor size was measured with calipers every 3 or 4 days. Upon completion of the tumor study, mice were euthanatized by isoflurane. Tumor volume was calculated as *ab*^2^/2 (mm^3^), where a and b are the largest and smallest central cross-sectional dimensions, respectively. At 26 days after the HSC-3 cell transplantation, tumors were extracted after the euthanasia treatment.

### Clinicopathological information

This clinical study using the patient’s information was done under the permission of the ethics committee in Fukuoka Dental College (# 312). The 36 cases (male/female: 24/12, mean age: 60.3 years (range: 27–88 years)) examined in this study consisted of 8 oral epithelial dysplasia (OED) (Histological grade; moderate: 5, severe and carcinoma in situ: 3) and 28 OSCC (Histological differentiation; well: 11, moderate: 10, moderate to poor and poor: 7). These Japanese patients underwent surgery at Fukuoka Dental College Hospital between 2009 and 2018. The patients were not prescribed chemotherapy or irradiation before surgery. The histological classification was performed according to the criteria of the “WHO Classification of Head and Neck Tumours” [15].

### Immunohistochemical procedure

Immunohistochemistry was performed as previously described [16]. Briefly, all surgical specimens were fixed in formalin and embedded in paraffin, then cut into 4 μm-thick sections for H.E. and immunohistochemical staining. For the antigen retrieval, the sections were autoclaved for 5 min at 121 °C in 0.01 M citrate buffer solution, pH 6.0. The sections were incubated with the primary antibodies (mouse anti-human NFAT5 antibody, 1:200; rabbit anti-human EGFR antibody, 1:200; rabbit anti-human DPAGT1 antibody, 1:200) at 4 °C for overnight. These sections were then incubated with biotin-labeled antibody (Agilent, Santa Clara, CA), followed by the streptavidin-biotin-peroxidase complex method. For the immunofluorescent histochemical staining, after the incubation with each primary antibody, the sections were treated with immunofluorescent labeled secondary antibodies at room temperature for 1 h. Sections were mounted using VECTASHIELD (Vector Lab., Burlingame, CA) and visualized at the appropriate wavelength using a fluorescence microscope (Axio Vert. A1, Carl Zeiss).

### Immunohistochemical assessment

We randomly chose three areas in each dysplastic or cancerous lesion in one case and counted the number of cells positive for nuclear NFAT5 or cytoplasm DPAGT1 in at least 300 dysplastic or cancer cells included in one chosen area. The degree of positivity of immunoreaction in each area was determined according to the modified method of the one originally described by Allred et al [17]. Briefly, the percentage of immunostaining-positive dysplastic or cancer cells was described as proportion score (PS) [scored on a scale of 0–3; 0: 0%, 1: <10% (NFAT5 and DPAGT1), 2: <30% (NFAT5) or 50% (DPAGT1), 3: equal or more than 30% (NFAT5) or 50% (DPAGT1)]. Staining intensity was also described as intensity score (IS) [scored on a scale of 0–3; 0: negative, 1: weak positive, 2: intermediate positive, 3: strong positive]. The proportion and IS were then summed to produce total score (TS) (TS = PS + IS) [scored on a scale of 0, 2–6].

### Statistical analyses

All data were expressed as the mean ± standard error of the mean (SEM). Student’s *t* test, Dunnett’s test, one-way analysis of variance and Mann–Whitney *U* test were used for statistical evaluations. Statistical significance was set as *p < 0.05, **p < 0.01 and ***p < 0.001.

## Results

### EGFR is translocated from the cytoplasm to the plasma membrane in OSCC cells under hyperosmotic conditions

**Fig. 1:**
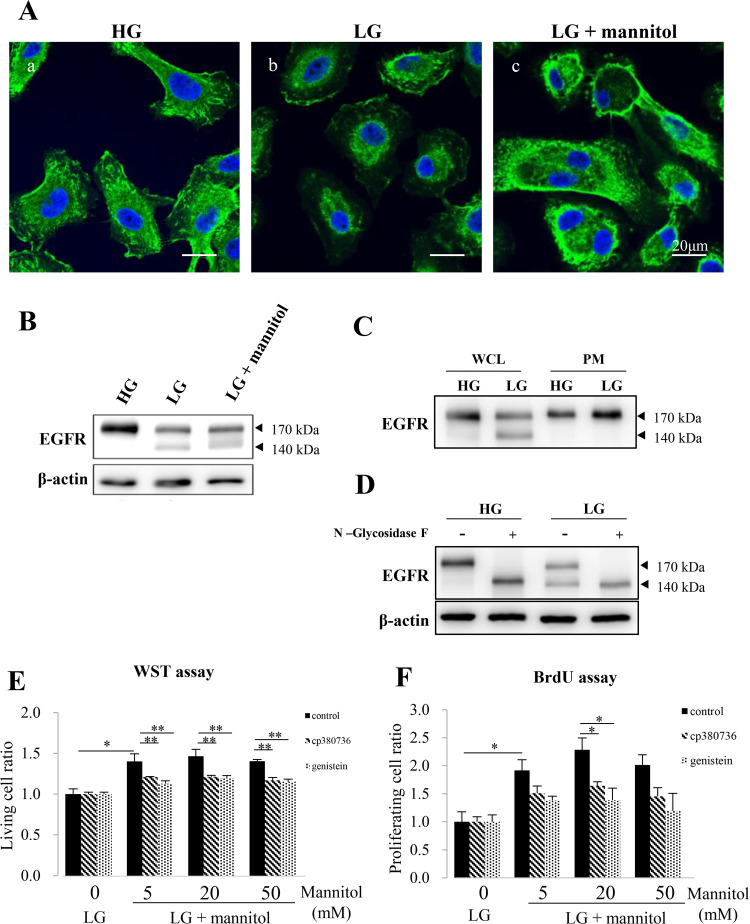
Changes in EGFR expression and subcellular localization in HSC-3 cells under hyperosmotic conditions. **a** Immunofluorescent cytochemical staining of EGFR (green) with nuclear counter staining (blue) for HSC-3 cells. Cells were cultured for 72 h in high glucose (HG), low glucose (LG) or LG with mannitol (LG + mannitol (50 mM)) medium and fixed with 4% PFA. EGFR is largely expressed in the plasma membrane of HSC-3 cells cultured in HG medium (HG). Conversely, perinuclear staining of EGFR is apparent in HSC-3 cells cultured in LG (LG). However, the immunolocalization of EGFR is shifted to the plasma membrane from the perinuclear cytoplasm in the LG + mannitol culture condition (LG + mannitol). Scale bars = 20 µm. **b** Western blot analysis for the expression of EGFR in HSC-3 cells. A 170 kDa band of EGFR was only detected when cells were maintained in a HG medium (HG). However, both a 170 kDa and a 140 kDa bands were detected when cells were maintained in a LG medium (LG). Moreover, when cells were cultured in a LG medium under the hyperosmotic condition, the 140 kDa band becomes obscure with a smearing staining in the region between 140 and 170 kDa molecular weights (LG + mannitol). **c** Western blots for the protein extracts from whole cell lysates (WCL) and plasma membrane fractions (PM) using an anti-EGFR antibody. A 140 kDa band is only detected in LG in WCL, suggesting that 140 kDa EGFR is localized in the perinuclear cytoplasm and 170 kDa EGFR is localized in the plasma membrane of HSC-3 cells together with the results of EGFR immunocytochemistry in (**a**). **d** Western blots for the protein extracts from HSC-3 cells cultured in HG or LG medium with (+) or without (−) treatment of N-glycosidase F. In the N-glycosidase F-treated condition, only a non-glycosylated 140 kDa band was found in HSC-3 cells cultured in both HG and LG mediums. This result reveals that 170 kDa EGFR is glycosylated and 140 kDa EGFR is non-glycosylated, and the smear EGFR (**b**; LG + mannitol) suggests the combined bands of different glycosylation states of EGFR. β-actin=Endogenous control. **e** Comparisons of the living cell ratio by WST assay. When cultured without the EGFR signaling inhibitors CP380736 and genistein, the live cell ratio in each LG + mannitol (5, 20 and 50 mM) is statistically higher than that in LG with no mannitol (black bars). Under the culture condition with each EGFR signaling inhibitor, the cell living is significantly inhibited in each LG + mannitol (5, 20 and 50 mM) (gray and light gray bars). **f** Comparisons of the cell proliferation by BrdU assay. Under the culture conditions without EGFR signaling inhibitors, the proliferation cell ratio in each LG + mannitol (5, 20 and 50 mM) is statistically higher than that in LG with no mannitol (black bars). Under the culture condition with each EGFR signaling inhibitor, the cell proliferation in each LG + mannitol (5, 20 and 50 mM) is also inhibited by EGFR inhibitors and the tendency is more apparent in the culture with genistein than in that with CP380736 although the statistically significant difference is only seen in LG + 20 mM mannitol (gray and light gray bars). The statistical significance assessed by Student’s *t* test is indicated by *p < 0.05 and **p < 0.01.

We first examined the subcellular localization of EGFR in HSC-3 cells cultured in either a HG or a LG medium. Immunofluorescent cytochemical staining revealed that EGFR was largely located in the plasma membrane when cells were cultured in a HG medium (Fig. 1a(a)). Conversely, EGFR was largely located in the cytoplasm when cells were cultured in a LG medium (Fig. 1a(b)). On the other hand, when cells were maintained under hyperosmotic conditions by the addition of mannitol, translocation of EGFR from the cytoplasm toward the plasma membrane was observed (Fig. 1a(c)). The same tendencies were observed in HSC-4 cells (Supplementary Fig. 1).

We next examined the expression of EGFR protein by Western blotting. When cells were maintained in a HG medium, EGFR was only detected as a band with an apparent molecular mass of 170 kDa. However, in a LG medium, we detected not only a 170 kDa band but also a 140 kDa band. Moreover, when cells were cultured in a LG medium under hyperosmotic conditions, a decrease in the 140 kDa band was observed (Fig. 1b). To examine which EGFR form came from which subcellular compartment, the cytoplasm or the plasma membrane, we extracted cell surface proteins and separated them by SDS-PAGE. Only the 170 kDa band was detected in the plasma membrane fraction of cells cultured in both the LG and HG medium (Fig. 1c). However, the 140 kDa band was detected in the proteins from whole cell lysates of cells cultured only in a LG medium.

To confirm if the differences between the two forms of EGFR was related to glycosylation, we employed N-glycosidase F, which cleaves carbohydrate chains of glycoproteins. With this tratment, only a 140 kDa band could be detected in both the LG and HG medium (Fig. 1d). These results indicate that the 170 KDa form of EGFR was a glycosylated form of 140 kDa EGFR found in the plasma membrane, while the 140 kDa form of EGFR was a non-glycosylation form found in the cytosol.

### EGFR signaling plays a key role in the acceleration of HSC-3 cell proliferation by hyperosmotic stimuli

To examine the effect of hyperosmolarity on the HSC-3 cell proliferation, we performed WST (water-soluble tetrazolium) and BrdU assays. The WST and the BrdU assay ratios were both upregulated by hyperosmotic stimuli (Fig. 1e, f). However, upregulation was inhibited by the treatment of CP380736, a specific EGFR inhibitor, or genistein, a nonspecific TKI (Fig. 1e, f) with both assays. These results indicate that EGFR signaling plays a key role in the acceleration of OSCC cell proliferation by hyperosmotic stimuli.

### NFAT is essential for the glycosylation of EGFR

**Fig. 2:**
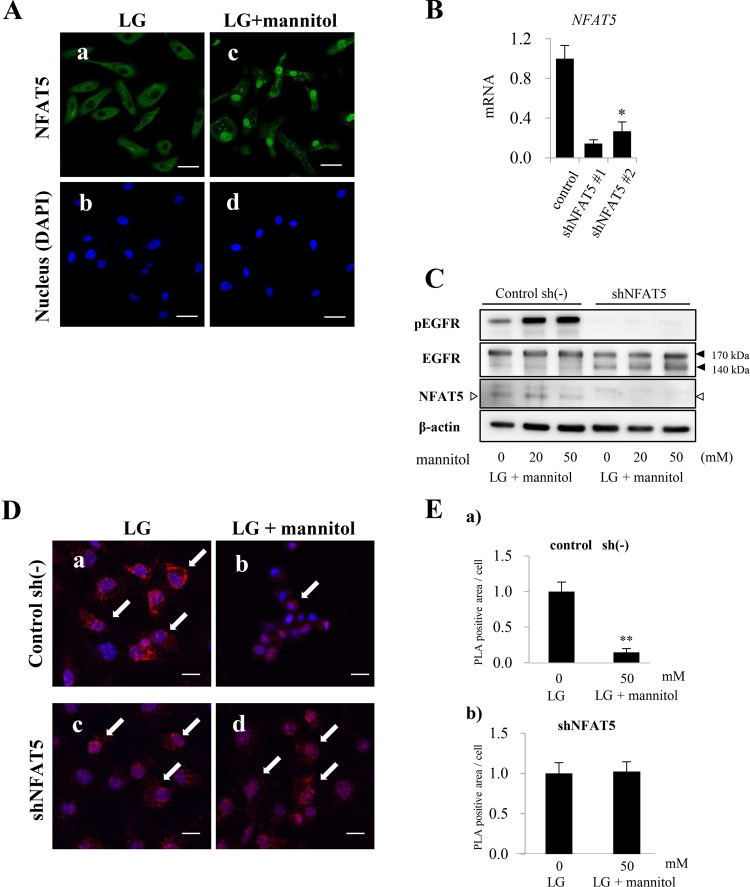
Translocation of NFAT5 into the nucleus activates EGFR phosphorylation and dissociation of pEGFR from the ER in HSC-3 cells under hyperosmotic conditions. **a** Immunofluorescent cytochemical staining of NFAT5 (green) for HSC-3 cells. Immunoreaction is predominantly seen in nucleus in LG + mannitol(c) compared to the cytosolic reaction in LG(a). Blue: Nuclear counter staining by DAPI. Scale bars=20 µm. **b** Preliminary inhibition analysis of NFAT5 mRNA expression with two different shRNAs specific for NFAT5 (shNFAT5s) on HSC-3 cells in LG by Real-time qPCR method. Both #1 and #2 shNFAT5s statistically repress NFAT5 mRNA expression compared to that in control. **c** Western blot analysis of phosphorylated EGFR (pEGFR), EGFR and NFAT5 expression in control sh(-) and NFAT5-silenced (shNFAT5) HSC-3 cells. The band equivalent for NFAT5 is distinct in control sh(-) cells in LG and LG + mannitol (20 and 50 mM) but indistinct in shNFAT5 cells in LG and LG + mannitol. In control sh(-) cells, the band of non-glycosylated 140 kDa EGFR is apparent in LG but indistinct in LG + mannitol. pEGFR expression is also detected in LG and is upregulated in LG + mannitol in control sh(-) cells. In contrast, in shNFAT5 cells, the expression of 140 kDa EGFR is apparent and no obvious change is seen in 170 kDa/140 kDa EGFR expression ratio in LG and LG + mannitol. In addition, expression of pEGFR is not distinct in LG and LG + mannitol in shNFAT5 cells. β-actin = Endogenous control. **d** In situ proximity ligation assay to confirm the effect of hyperosmolarity. Colocalization of EGFR and KDEL which are secondary labeled by PLA probes (red dots; arrow) is apparent in both control sh (-) (**a**) and shNFAT5 (**c**) cells in LG, and also in shNFAT5 cells in LG + mannitol(d). However, the co-localization is indistinct in control sh (-) cells in LG + mannitol(b). Blue: Nuclear counter staining by DAPI. Scale bars = 20 µm. **e** The bar graphs indicate quantitative values of PLA signals. The signal is statistically lower in LG + mannitol (50 mM) in control sh (-) cells (**a**). The difference is diminished in LG + mannitol in shNFAT5 cells (**b**). Together with the results in **d**, it is considered that NFAT5 dissociates colocalization of EGFR and KDEL in control sh (-) cells in LG + mannitol. Statistical significance assessed by Student’s *t* test is indicated by *p < 0.05 and **p < 0.01.

NFAT5 has been reported to play a pivotal role in gene expression in response to extracellular hyperosmotic conditions. In our analyses, NFAT5 expression was observed in the cytoplasm of HSC-3 cells. However, NFAT5 expression was found in the nucleus of HSC-3 cells in the hyperosmotic culture condition (Fig. 2a; green in a and c). The same tendencies were observed in HSC-4 cells (Supplementary Fig. 2A).

To determine the effect of the change of the NFAT5 subcellular localization under hyperosmotic conditions on EGFR expression, we depleted NFAT5 from HSC-3 cells using short hairpin RNA against *NFAT5* (shNFAT5). We first confirmed that shNFAT5 significantly repressed the *NFAT5* expression (Fig. 2b). In HSC-3 cells cultured with no shNFAT5 treatment, the expression of phosphorylated EGFR (pEGFR) was observed under normal osmotic conditions and was increased with the hyperosmotic stimuli (Fig. 2c, pEGFR in Control sh(-)). On the other hand, no pEGFR expression was observed in *NFAT5*-silenced cells under the normal osmotic and hyperosmotic stimuli conditions (Fig. 2c, pEGFR in shNFAT5). In addition, in accordance with the increase of hyperosmotic stimuli, the expression of 140 kDa EGFR under normal osmotic conditions was decreased with sh(-) culture condition but was not decreased with the treatment of shNFAT5 culture condition. (Fig. 2c, EGFR in Control sh(-)). In contrast, in *NFAT5*-silenced cells, the expression of 140 kDa EGFR was observed but no significant changes were observed with hyperosmotic stimuli (Fig. 2c, EGFR in shNFAT5).

To confirm the difference between the 140 and 170 kDa EGFR, we focused on the glycosylation status of EGFR. Protein glycosylation is initiated in the endoplasmic reticulum (ER), we examined the proximity of EGFR and ER by in situ PLA. In this assay, co-localization of two proteins was visualized as fluorescent signals. In HSC-3 cells cultured with no shNFAT5 treatment, we observed decreases of fluorescent signals upon hyperosmotic stimuli (Fig. 2d(a, b), e(a)). On the other hand, in NFAT5-silenced HSC-3 cells, a decrease in fluorescent signals was not observed by hyperosmotic stimuli (Fig. 2d(c, d), e(b)). These results indicate that NFAT5 is essential for the glycosylation of EGFR in ER.

### NFAT5 increases the expression of DPAGT1 by binding to the promoter region under hyperosmotic conditions

**Fig. 3:**
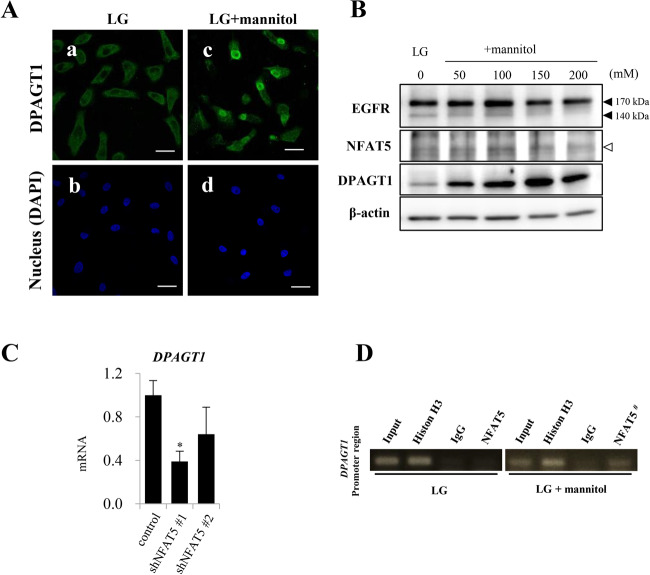
Expression and subcellular localization of DPAGT1 in HSC-3 cells under hyperosmotic condition. **a** Immunofluorescent cytochemical staining of DPAGT1 (green) in HSC-3 cells. Cytoplasmic expression is seen in in LG(a), but perinuclear expression is distinct in LG + mannitol(c). Blue: Nuclear counter staining by DAPI. Scale bars = 20 µm. **b** Western blot analysis of EGFR, NFAT5 and DPAGT1 in HSC-3 cells. DPAGT1 expression is seen in LG and becomes stronger in accordance with the increase of hyperosmolarity. Conversely, 140 kDa EGFR expression becomes obscure in accordance with the increase of hyperosmolarity and DPAGT1 expression. No obvious change is seen in NFAT5 expression in LG and LG + mannitol. β-actin = Endogenous control. **c** Real-Time qPCR analysis of DPAGT1 mRNA expression in HSC-3 cells. #1 shNFAT5 repress DPAGT1 mRNA expression with a statistical significance. Same tendency but with no statistical significance is seen in the inhibition assay using #2 shNFAT5. **d** Chromatin immunoprecipitation (ChIP) assay to clarify NFAT5 binding to DPAGT1 promoter region in HSC-3 cells. Co-immunoprecipitation of NFAT5 with DPAGT1 promoter region is seen as a band with anti-NFAT5 antibody immunoreaction in LG + mannitol(50 mM) (NFAT5#). Anti-Histon H3 and IgG antibodies are used for positive and negative control, respectively. Statistical significance assessed by Student’s *t* test is indicated by *p < 0.05.

We hypothesized that glycosylation of EGFR in ER is important for cell proliferation. We examined some regulators involved in N-glycosylation and focused on DPAGT1, which is the first enzyme in the pathway for protein N-glycosylation. We analyzed DPAGT1 expression and found that hyperosmotic stimuli induced and increased the expression of DPAGT1 in accordance with the increase of hyperosmotic stimuli (Fig. 3a, b). The same trend was observed in HSC-4 cells (Supplementary Fig. 2B). Conversely, the expression of 140 kDa EGFR was decreased with the increase of hyperosmotic stimuli (Fig. 3b). Moreover, RT-qPCR analyses revealed that *NFAT5* silencing by two different shNFAT5s reduced mRNA levels of *DPAGT1* (Fig. 3c).

We performed ChIP analyses to assess whether NFAT5 was bound to the *DPAGT1* promoter region, and found that NFAT5 bound to the *DPAGT1* promoter region in HSC-3 cells cultured under hyperosmotic conditions (Fig. 3d). These results suggest that hyperosmotic stimuli upregulate the expression of DPAGT1 by the activation of NFAT5.

### Tumorigenicity of HSC-3 cells can be accelerated, depending on hyperosmotic stress in vivo

**Fig. 4:**
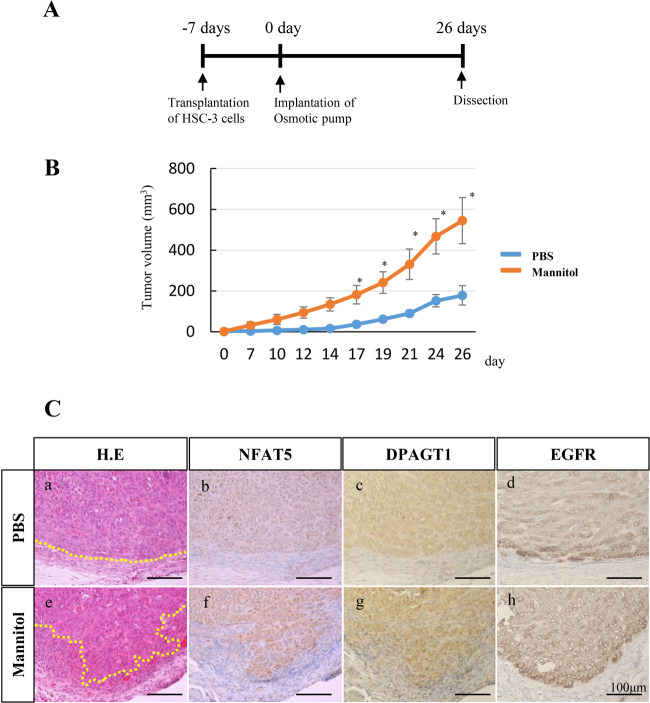
In vivo analyses of tumorigenicity and biological changes in transplanted HSC-3 cells in hyperosmotic stress-induced tumor microenvironments. **a** A scheme of the in vivo experimental protocol. HSC-3 cells are transplanted into the right flank of the 8-week-old female BALB/cAJcl-nu mice. Osmotic pumps are implanted seven days after the HSC-3 cell transplantation. **b** Tracing and comparison of daily changes of tumor volumes between the control (PBS; *n* = 6) and hyperosmotic stress-induced (Mannitol; *n* = 6) groups. Statistical significance assessed by Dunnett’s test is indicated by *p < 0.05. **c** Tumor histologies at the 26th day after the HSC-3 cell transplantation. The upper (a-d) and lower (e-h) columns reveal the results from the control (PBS) and hyperosmotic stress-induced (Mannitol) groups, respectively. H.E. staining reveals that the tumor shows an invasive growth and an irregular tumor margin in the Mannitol group (e) while the tumor shows an expansive growth and a smooth tumor margin in the PBS group (a). Yellow dot lines indicate tumor margins in PBS (a) and Mannitol (e) groups. Immunohistochemical analyses reveal that expression of NFAT5, DPAGT1 and EGFR are all stronger in the Mannitol group (f, g, h) than those in the control group (b, c, d). Scale bars=100 µm.

We investigated the effect of hyperosmotic stress on the tumorigenicity of HSC-3 cell xenografts using BALB/cAJcl-nu mice (Fig. 4a). HSC-3 cells were injected into the right flank of the mice. After 1 week from the injection, a microosmotic pump was implanted in the right flank to sustain the release of PBS or mannitol into the microenvironment of the transplanted HSC-3 cells. HSC-3 tumorigenicity was significantly accelerated depending on hyperosmotic stress induced by the sustained release of mannitol (Fig. 4b). Histopathologically, tumors showed prominent invasive growth at the tumor front in the hyperosmotic stress group (Fig. 4c(e)). On the other hand, tumors of the PBS control group reveled an expansive growth with a clear margin at the tumor front (Fig. 4c(a)). Immunohistochemically, NFAT5 and DPAGT1 expression were both upregulated in the tumor cells of the hyperosmotic stress group (Fig. 4c(f, g)) compared to those in the tumor cells of the PBS control group (Fig. 4c(b, c)). The membranous expression of EGFR was also upregulated in the tumor cells of the hyperosmotic stress group (Fig. 4c(h)) compared to those in the tumor cells of the control group (Fig. 4c(d)).

### Immunohistochemical analysis of NFAT5, DPAGT1, and EGFR expression in OSCC in vivo

**Fig. 5:**
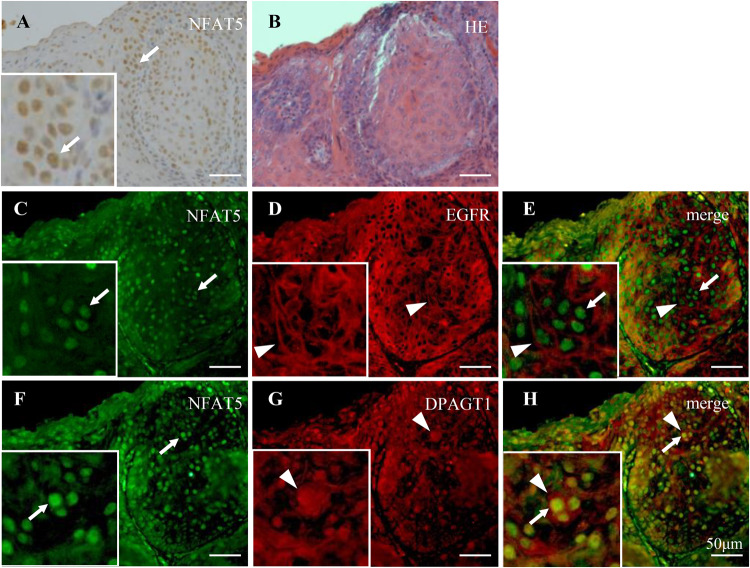
Immunohistochemical analyses of NFAT5, EGFR and DPAGT1 expression in human oral squamous cell carcinomas. Solid nests of a human tongue squamous cell carcinoma (**b**: H.E.) are applied for immunohistochemical analyses with anti-NFAT5 (**a**: Brown, **c**, f: Green), -EGFR (**d**: red) and -DPAGT1 (**g**: red) antibodies. Merged images are shown as NFAT5 and EGFR (**e**), and NFAT5 and DPAGT1 (**h**). These immunohistochemical analyses reveal the nuclear localization of NFAT5 (arrows in **a**, **c**, **e**, **f**, **h**), cell surface localization of EGFR (arrowheads in **d**, **e**) and perinuclear localization of DPAGT1 (arrowheads in **g**, **h**). Each inset is an enlargement of the region directed by an arrow or arrowhead in each figure. Scale bars = 50 µm.

**Fig. 6:**
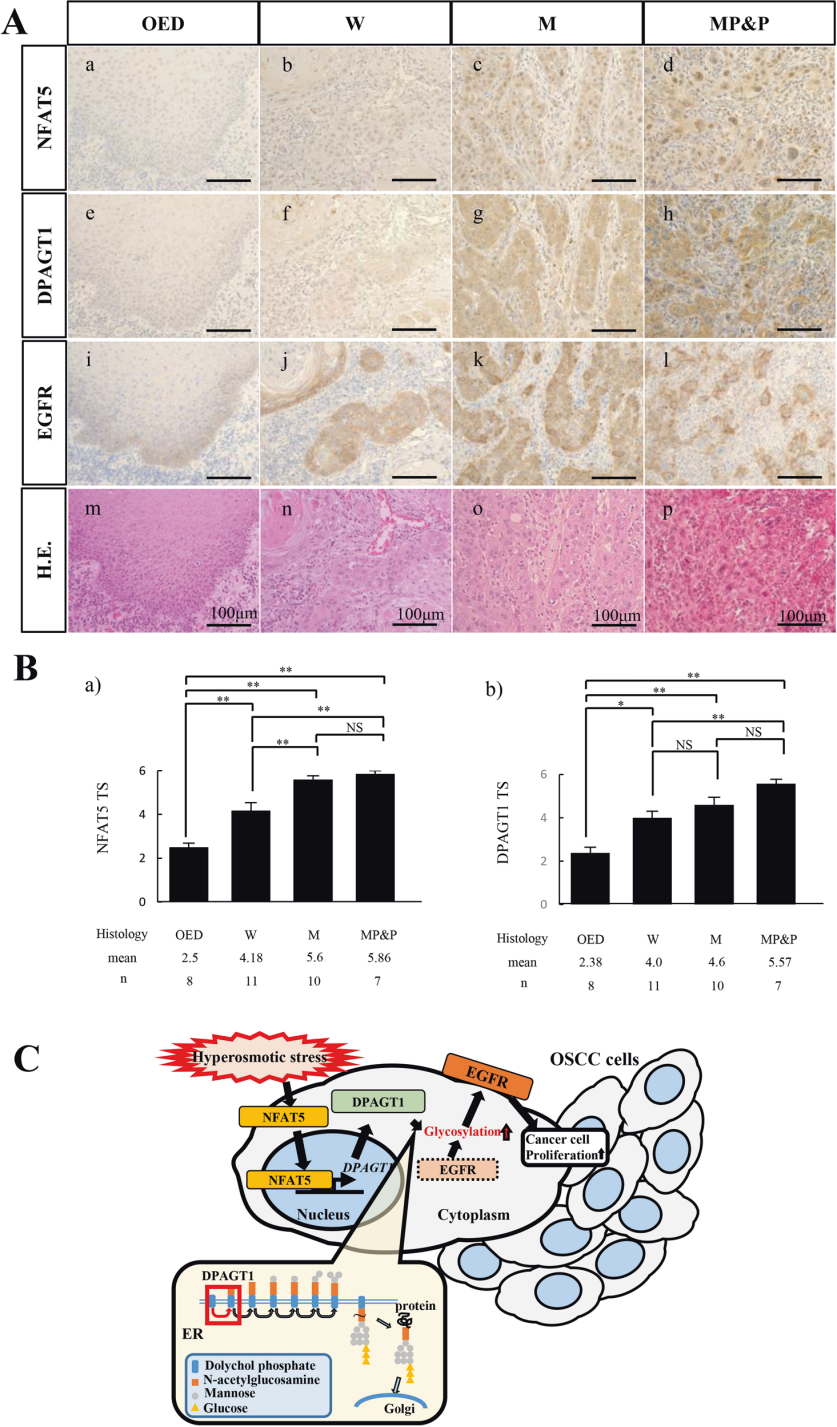
Immunohistochemical analyses of NFAT5, DPAGT1 and EGFR expression in oral epithelial dysplasia (DP) and degree of oral squamous cell carcinoma (OSCC). **a** NFAT5 (a), DPAGT1 (e) and EGFR (i) expression are all week in cases of oral epithelial dysplasia (OED), and each expression becomes strong in accordance with the advanced histological differentiation degree of OSCCs as is shown in the well differentiated (W) (b, f, j), moderately differentiated (M) (c, g, k), and moderately to poorly and poorly differentiated (MP&P) (d, h, l) cases. H.E. staining is shown in the lowest column (m-p). Scale bars = 100 μm. **b** Bar graphs indicate comparisons of NFAT5 (a) and DPAGT1 (b) TS scores in OED and each histological differentiation degree of OSCC cases (*n* = 8 (OED), 11 (W), 10 (M) and 7 (MP&P)). TS scores of NFAT5 and DPAGT1 expression are both higher in OSCC than in OED cases with a statistical significance. In addition, both TS scores become higher in accordance with the advanced histological differentiation degree of OSCCs although the statistical significance is only seen in the NFAT5 TS score between W and M OSCC cases. The statistical significance assessed by Mann–Whitney *U* test is indicated by *p < 0.05 and **p < 0.01. **c** The illustrated schematic explains the mechanism representative of the induced proliferative activity of OSCC cells by hyperosmotic stress based on the results in this study.

We immunohistochemically analyzed the distribution of NFAT5, EGFR, and DPAGT1 protein expression in human tongue OSCC specimens. Well to moderately differentiated OSCC cells were clearly stained with an anti-NFAT5 antibody in their nuclei (Fig. 5; arrows in a, c and f, and merge e and h). EGFR and DPAGT1 were expressed on the cancer cell surface (Fig. 5; arrowheads in d and merge e) and in the perinuclear area (Fig. 5; arrowheads in g and merge h), respectively. Next, these expression were analyzed in the precancerous lesion of OED and well (W), moderately(M), and moderately to poorly and poorly (MP&P) differentiated OSCCs. The NFAT5 intra-nuclear expression was weak or not apparent in OED (Fig. 6a(a)), but apparent in each differentiation degree of OSCC (Fig. 6a(b–d)). The strong intra-nuclear expression of NFAT5 was seen especially in the invasive and poorly differentiated cancer cells in M and MP&P OSCCs (Fig. 6a(c, d)). Similarly, DPAGT1 expression was weak or not apparent in OED (Fig. 6a(e)), but apparent in each differentiation degree of OSCC (Fig. 6a(f–h)). Furthermore, the membranous expression of EGFR was apparent in accordance with the strength of the NFAT5 intra-nuclear and DPAGT1 cytoplasmic/perinuclear expression (Fig. 6a(i–l)). Finally, expression total scores (TSs) were compared among OED and each differentiation degree of OSCC by as described in materials and methods. TSs of NFAT5 and DPAGT1 expression in each differentiation of OSCC were significantly higher than those in OED, and TS in OSCC was gradually increased in accordance with the poor differentiation. A statistical difference was seen between W and MP&P in each NFAT5 and DPAGT1 expression (Fig. 6b(a, b)).

## Discussion

In the cancer microenvironment, cancer cells are exposed to starvation, hypoxia and solid stress, and these factors impact on cancer progression [18–21]. In general, the cancer microenvironment is constantly inflamed and inflammatory responses against cancer cells play an important role in cancer development [7, 8]. Previous researchers have studied the relationship between inflammatory cytokines and cancer cell progression in the cancer microenvironment, but osmolarity is also elevated in the cancer microenvironment. Cancer cells are constantly undergoing apoptosis and necrosis especially in the center of the cancer cell nests, because hypoxia and starvation occur due to the low resorption of oxygen. Intracellular potassium ions are then released from the apoptotic or necrotic cancer cells into the extracellular fluid, resulting in the elevation of osmolarity [22]. However, the role of hyperosmotic stress on cancer cell proliferation is not fully understood. In this study, we found that NFAT5 was upregulated in OSCC cancer cells in association with the upregulation of DPAGT1. In addition, in vitro studies using HSC-3 cells revealed that hyperosmotic stress upregulated cell proliferation and NFAT5 expression which resulted in changes in the subcellular localization of EGFR from the cytoplasm to the plasma membrane.

Cancer cells display a high rate of glycolysis rather than oxidative phosphorylation for energy production. Therefore, glucose utilization of cancer cells is greatly enhanced [23, 24]. Consequently, it is thought that glucose levels in the microenvironment surrounding tumor cells are decreased [19, 25]. A previous report revealed that glucose concentrations were much lower in tumor than normal tissue in colon and stomach cancer [25]. To mimic this condition, we first cultured cancer cells in LG medium, and immunocytochemically observed EGFR expression predominantly in the cytoplasm, especially in the perinuclear region. In addition, we detected unglycosylated EGFR of 140 kDa which is not observed in the plasma membrane fraction by Western blotting analyses. Next, we investigated EGFR expression in HSC-3 cells under hyperosmotic conditions. To mimic hyperosmotic conditions, cells were cultured in LG medium with mannitol, which is an impermeable material. When cells were cultured under hyperosmotic conditions, we observed the EGFR expression predominantly in the plasma membrane and detected a glycosylated form of 170 kDa; whereas the unglycosylated EGFR (140 kDa) was faint by Western blot analyses. These results indicate that glycosylation of EGFR was essential for its subcellular translocation from the cytoplasm to the plasm membrane under hyperosmotic conditions. In addition, the addition of NaCl has been also known to elevate the osmolarity of medium, and we also observed the same finding of the subcellular EGFR translocation from the cytoplasm to the plasma membrane when cells were cultured in medium with NaCl (data not shown). Furthermore, upregulation of cancer cell proliferation was observed in accordance with the increase of the osmolarity of medium, and was then suppressed by CP380736, a specific EGFR inhibitor, and genistein, a nonspecific TKI. Thus, these results indicate that not only glucose concentration but also osmolarity in the cancer microenvironment were critical factors for the enhancement of the EGFR signaling pathway in cancer cell progression.

NFAT5 is a transcription factor activated under hyperosmotic conditions and was initially reported to play a role in orchestrating a genetic program to restore cellular homeostasis in the hypertonic kidney inner medulla. When cells are exposed to osmotic stress, NFAT5 activates transcription of several osmoadaptive genes to restore biochemical homeostasis [26]. Some previous studies showed activation of NFAT5 in colon, renal, and breast cancer [27–29]. To investigate the role of NFAT5 in OSCC, we knocked down the gene using short hairpin RNA. Interestingly, the translocation of EGFR from ER toward plasma membrane and glycosylation of EGFR in hyperosmotic stress were significantly inhibited by shRNA for NFAT5 in HSC-3 OSCC cells. In addition, RT-qPCR analysis revealed that DPAGT1 gene expression was suppressed by knockdown of NFAT5. From these findings, it appears that EGFR subcellular translocation and glycosylation was triggered at least by the activation of NFAT5.

To clarify that NFAT5 directly regulated the DPAGT1 upregulation in HSC-3 cells by binding the promoter region of DPAGT1, we performed a ChIP assay. The assay revealed that NFAT5 protein bound to the DPAGT1 promoter region in HSC-3 cells under hyperosmotic conditions. The DPAGT1 gene encodes dolichol phosphate-dependent N-acetylglucosamine-1-phosphate transferase, which catalyzes the initial reaction required for synthesis of dolichol-P-P-oligosaccharides in the ER [30]. Previous studies reported that DPAGT1 gene and proteins were highly expressed in OSCC, and regulated cancer cell proliferation [31–33]. Nita-Lazar et al. reported that over expression of DPAGT1 induced aberrant glycosylation of E-cadherin and discohesion in oral cancer cells, leading to the induction of cancer cell migration [31]. It is reasonable to propose that DPAGT1 plays a pivotal role in both cell proliferation and migration. In a murine melanoma in vivo model, Kim et al. demonstrated that NFAT5 was critical for melanoma cell proliferation and metastasis [34]. In our analyses, NFAT5 and DPAGT1 protein expression was upregulated in the cells of the xenografts under a hyperosmotic cancer microenvironment, and contributed to the enhancement of the progression and invasion of cancer cells of the tumor mass formed by transplanted HSC-3 cells. These findings indicate that NFAT5 may play a pivotal role in the enhancement of the progression of OSCC. We also observed distinct perinuclear and nuclear immunoreactions of DPAGT1 and NFAT5 proteins in human OSCC cells, respectively. In addition, both expression TS scores were increased in accordance with the increased histological degrees of OSCC. Overall, these results demonstrate that hyperosmotic stress contributed to the activation of DPAGT1 and the subcellular translocation of EGFR via NFAT5 induction in the tumor microenvironment (Fig. 6c).

In summary, we determined that NFAT5 enhanced the progression of OSCC cells under hyperosmotic conditions by activating DPAGT1 and the glycosylation of EGFR, which resulted in the a change of EGFR subcellular localization from the cytoplasm to the plasma membrane. Our present study may provide a new insight into a potential therapeutic target for OSCC.

## Supplementary information

Supplemental Figures
