# Supplementary material for: NFAT5 promotes oral squamous cell carcinoma progression in a hyperosmotic environment
Source: Lab Invest. 2020 Sep 8;101(1):38–50. doi: 10.1038/s41374-020-00486-1 (PMC7758185; doi:10.1038/s41374-020-00486-1)
Supplement: Supplementary file 1 — Supplemental Figures [file 41374_2020_486_MOESM1_ESM.pdf]

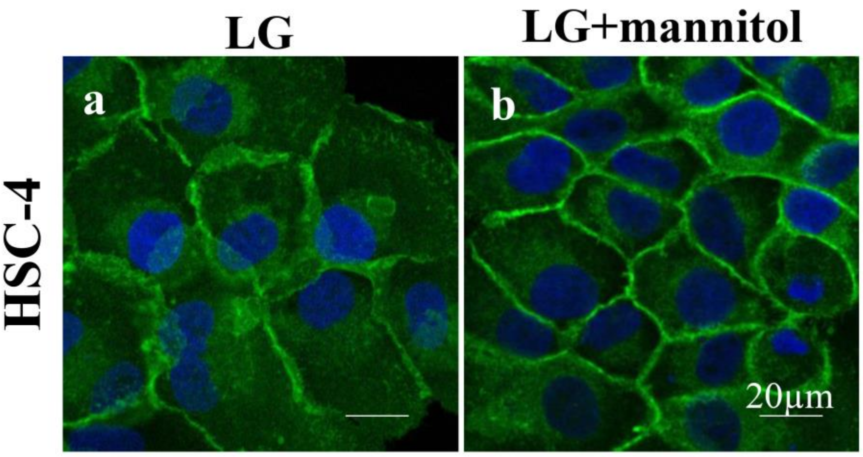

Supplemental Figure 1. Expression and subcellular localization of EGFR in HSC-4 cells in the normo- and hyper-osmotic conditions.

Immunofluorescent cytochemical staining of EGFR (green) with nuclear counter staining by DAPI (blue) for HSC-4 cells (a, b). Cells were cultured for 72hs in the Low Glucose (LG) or Low Glucose with mannitol (50mM) (LG + mannitol) culture conditions and fixed with 4% PFA. The perinuclear staining of EGFR is distinct in HSC-4 cells cultured in LG (a). However, the immunolocalization of EGFR is largely seen in the plasma membrane and the perinuclear staining of EGFR is indistinct in HSC-4 cells cultured in LG + mannitol (b). Scale bars = 50 μm.

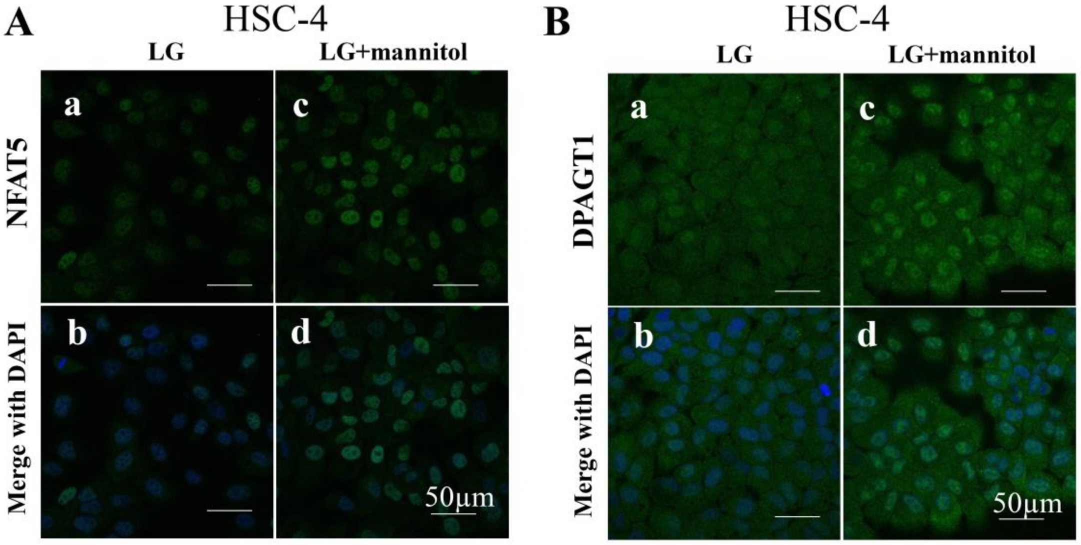

Supplemental Figure 2. Expression and subcellular localization of NFAT5 and DPAGT1 in HSC-4 cells in the normo- and hyper-osmotic conditions.

A. Immunofluorescent cytochemical staining of NFAT5 (green) for HSC-4 cells. Strong immunoreaction of NFAT5 is seen predominantly in the nuclei in LG+mannitol (c, d), but only weak nuclear or cytosolic immunoreaction is seen in LG (a, b). B. Immunofluorescent cytochemical staining of DPAGT1 (green) in HSC-4 cells. Perinuclear expression is distinct in LG+mannitol (c, d) but is indistinct in LG (a, b). Blue (b and d in A, B): Nuclear counter staining by DAPI. Scale bars = 50μm.
